# Increased NK Cell Count in Multiple Sclerosis Patients Treated With Dimethyl Fumarate: A 2-Year Longitudinal Study

**DOI:** 10.3389/fimmu.2019.01666

**Published:** 2019-07-19

**Authors:** Damiano Marastoni, Alessandro Buriani, Anna Isabella Pisani, Francesco Crescenzo, Carmela Zuco, Stefano Fortinguerra, Vincenzo Sorrenti, Bruno Marenda, Chiara Romualdi, Roberta Magliozzi, Salvatore Monaco, Massimiliano Calabrese

**Affiliations:** ^1^Neurology B, Department of Neurosciences, Biomedicine and Movement Sciences, University of Verona, Verona, Italy; ^2^Data Medica Group, Maria Paola Belloni Center for Personalized Medicine, Synlab Limited, Padova, Italy; ^3^Department of Biology, University of Padova, Padova, Italy

**Keywords:** multiple sclerosis, dimethyl fumarate, lymphopenia, natural killer cells, cell count, lymphocyte subsets

## Abstract

**Background:** Dimethyl fumarate (DMF) is a disease-modifying drug for relapsing-remitting multiple sclerosis. Among others, DMF impedes immune activation by shifting the balance between inflammatory and regulatory cell types and by inducing apoptosis-triggered lymphopenia. Although the decrease in lymphocyte count is an early effect of the drug in several patients, the long-term impact on lymphocyte subsets is largely unknown.

**Methods:** We performed a 2-years observational study on total lymphocyte count and subsets thereof by flow cytometry of peripheral blood of 38 multiple sclerosis patients in treatment with DMF. Data were collected at the beginning and after 3, 6, 12, and 24 months of therapy.

**Results:** Total lymphocyte count decreased in relation to time of exposure to DMF. Mean absolute B cell count decreased by 34.1% (*p* < 0.001) within the first 3 months of therapy and then remained stable over time. Mean absolute CD3^+^ T cells count decrement reached 47.5% after 12 months of treatment (*p* < 0.001). NK cells count showed a heterogeneous trend, increasing by 85.9% (*p* < 0.001) after 2 years of treatment. CD4^+^ T cells and CD8^+^ T cells substantially decreased, with a significant increase of CD4^+^/CD8^+^ ratio during the first year of therapy.

**Conclusions:** NK cells showed a heterogeneous behavior during DMF treatment with a significant increase over time. Since NK cells may also have a regulatory effect on immune system modulation, their increase during DMF treatment might play a role in the efficacy and safety of the drug.

## Background

Dimethyl fumarate (DMF) is an approved first-line disease-modifying treatment for relapsing remitting multiple sclerosis (RRMS). DMF has multiple mechanisms of action (1), including activation of the neuroprotective nuclear factor erythroid-derived 2-related factor 2 (Nrf2) transcriptional pathway (2) and modulation of nuclear factor kB pro-inflammatory pathway (3). Recently, DMF and its active metabolite monomethyl fumarate (MMF) have been shown to target and inactivate the glycolytic enzyme glyceraldehyde 3-phosphate dehydrogenase (GAPDH) thus down-regulating aerobic glycolysis in myeloid and lymphoid cells and preventing immune activation (4). DMF treatment reduces the number of distinct T and B-cell subsets, even in the absence of prolonged and severe lymphopenia (5–9). Moreover, DMF therapy has been observed to increase the proportion of immune-regulatory CD56^bright^ NK cells in the first months of treatment (10), thus possibly influencing disease activity (11, 12).

Here we present an observational study aimed at investigating the long-term effects of DMF on different lymphocyte subsets in a cohort of relapsing-remitting multiple sclerosis patients.

## Methods

### Patients Cohort

This 2-years observational study included 38 patients aged from 22 to 58 years (mean age 38.0 ± 8.7 years, female sex 60.5%), with diagnosis of RRMS according to revised McDonald criteria. Patients were recruited at the Veneto Regional Multiple Sclerosis Center at University Hospital of Verona and were assigned to receive DMF at standard dose. Patients were examined every 3 months during the first year of treatment and every 6 months during the second year, with additional visits in case of relapses. A relapse was defined as a worsening of neurological impairment or appearance of a new symptom or abnormality attributable to MS, lasting at least 24 h and preceded by stability of at least 1 month (13). Expanded Disability Status Scale (EDSS) score (14) was assessed at each visit. Intercurrent infections were excluded by clinical and serological analyses. Twenty-five patients were treatment naïve; 13 patients had one (6) or more (7) previous treatments. Last treatments before introducing DMF were interferon beta-1a (9), interferon beta-1b (1), teriflunomide (1), glatiramer acetate (1), and cyclophosphamide (1) and were discontinued at least 3 months before initiating DMF therapy.

Patients with no evidence of disease activity (NEDA) or with evidence of disease activity (EDA) were also identified. NEDA was defined as a composite score obtained from three related measures of disease activity: (i) no evidence of relapses; (ii) no confirmed disability progression as assessed by an increase of the EDSS score by at least 1 point sustained over 6 months; and (iii) no new or newly enlarging white matter T2 lesions at the MRI follow-up (15).

The Local Ethic Committee approved the present study. Informed consent was obtained from all patients.

### Flow Cytometry

Complete blood count including absolute lymphocyte counts (ALCs) and count of total CD3^+^ T cells, CD19^+^ B cells and CD3^−^CD56^+^ NK cells was performed by flow cytometry by mean of ADVIA 2120i Hematology analyzer (16) at baseline and at 3, 6, 12, and 24 months after initiating DMF therapy (Supplementary Figure 3). Blood was processed immediately after the collection. Data about CD8^+^ T cells, CD4^+^ T cells, CD195^+^ T-helper 1 (Th-1), CD194^+^ T-helper 2 (Th-2), CD161^+^ T-helper 17 (Th-17) cells, and CD25^+^FOXP3^+^ T-regulatory (T-reg) were also obtained in a cohort of 13 patients. ALCs were graded in accordance with the Common Terminology Criteria for Adverse Events (CTCAE version 4) as follows: grade 0 (≥910 × 10^9^/L); grade 1 (≥800 × 10^9^/L); grade 2 (<800–500 × 10^9^/L); grade 3 (<500–200 × 10^9^/L), and grade 4 (<200 × 10^9^/L).

### MRI Analysis

Each patient underwent brain 3T MRI (at baseline, after 1 year and 2 years of clinical observation). MRI sequences were acquired by Philips Achieva 3T MR Scanner (Philips Medical Systems, Best, Netherlands). No software updating was carried out during the study period and the scanner underwent specific functioning test every 2 weeks to assure parameter stability and, thus, image reproducibility and comparability. For each subject 3D T2 weighted, 3D T1 weighted Fast Field Echo (FFE) and 3D Fluid Attenuated Inversion Recovery (FLAIR) image sets were acquired. Subjects were positioned for serial measurements according to published guidelines for serial MRI studies in MS (17).

### Statistical Analysis

Statistical analysis was performed using Prism 7.0 (GraphPad Software, La Jolla, CA) and R studio (Version 1.0.153); Wilcoxon matched-pairs signed-rank test for longitudinal analysis were applied; *p* < 0.05 were considered statistically significant. Linear regression was applied to evaluate the longitudinal rate of change in lymphocyte subsets. In the linear regression model, for each subject time and cell counts were used, respectively, as independent and dependent variables. Patients treated with DMF who had a positive longitudinal trend showed a positive β regression coefficient while patients who had a negative trend showed a negative β regression coefficient: higher the β coefficients, higher was the degree of increase or decrease of cell counts. An F-test was computed on the β regression coefficients obtained from linear regression to test the trend variability between NK cells and the other lymphocyte subsets. Mann-Whitney test was used to compare patients the EDA and NEDA groups for each lymphocyte subset both at the end of the first year of DMF treatment and at the end of the follow-up. A False discovery rate (FDR) correction was applied.

## Results

### Total Lymphocyte Count

**Figure 1:**
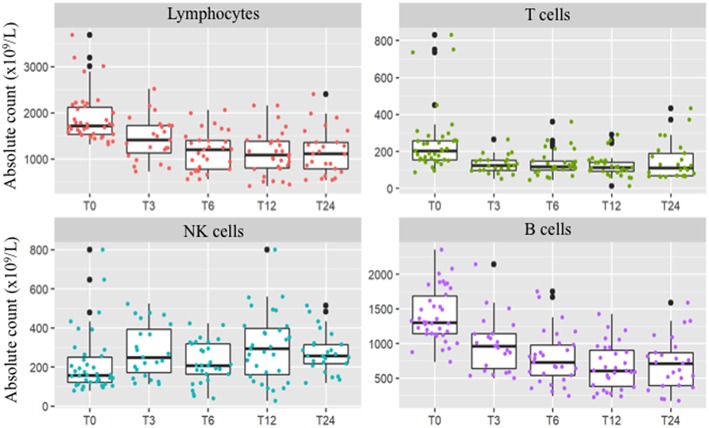
Peripheral absolute values [× 10^9^/L] of total lymphocyte count and T-cells, B-cells, and NK cells at baseline and after 3 (T3), 6 (T6), 12 (T12), and 24 (T24) months of DMF treatment. After 2 years of treatment total lymphocyte count persisted significantly decreased (*p* < 0.001). B and T-cells decrement was significant since the three months follow-up and was confirmed at the two year longitudinal analysis (*p* = 0.015 and *p* < 0.001, respectively). Conversely, NK cell count showed a different trend, with a significant increase at the 2 years follow-up (*p* < 0.001).

As expected, mean lymphocyte absolute count decreased during the 2 years of treatment (see Supplementary Table 1 for detailed absolute values at baseline and percentage changes at each time point). After 12 months of DMF exposure 2 patients (6.5%) developed persistent grade 3 lymphopenia, leading to discontinuation of the treatment (Figure 1); in addition, 4 patients (12.9%) had grade 2 lymphopenia, and 2 patients (6.5%) showed grade 1 lymphopenia. After 24 months of treatment, 1 patient (3.8%) had absolute count <500 × 10^9^/L, while 8 patients (30.8%) had total lymphocyte count between 500 and 800 cells × 10^9^/L (Figure 1). This trend was reflected by a reduced proportion of lymphocytes within leukocytes (not shown).

### T and B Cells

The mean absolute CD3^+^ T cells count progressively decreased during DMF exposure. Indeed, a 35% decrease was observed after 6 months of therapy, reaching 47.7% at the end of the second year of treatment (Supplementary Table 1). Among CD3^+^ T cells, CD8^+^ T lymphocytes showed a significant decrement after 1 year of treatment. In the second year the trend changed, as shown by changes of CD4^+^/CD8^+^ ratio (Table 1). Absolute CD8^+^ T cell values slightly increased during the second year of treatment (*p* = 0.021 when compared to 1-year follow up, Supplementary Figure 1) although remaining significantly lower than baseline values (*p* = 0.006). CD4^+^ T cells showed a downward trend, which did not reach statistical significance over the 2 years of follow-up (*p* = 0.080, Table 1).

**Table 1:** Absolute values [× 10^9^/L] and SD of T-cell subsets after 6, 12, and 24 months of DMF treatment compared to baseline values in a longitudinal analysis of a smaller cohort of patients.

	**Baseline** **(*n* = 13)**	**6 months** **(*n* = 13)**	**12 months** **(*n* = 13)**	**24 months** **(*n* = 13)**
CD4^+^ T	623.2 (212.0)	506 (245.4) *p* = 0.127	522 (282.0) *p* = 0.147	503.2 (282.0) *p* = 0.080
CD8^+^ T	368.6 (337.3)	237.5 (150.4) ***p*** **= 0.020**	150.7 (88.0) ***p*** **= 0.002**	196 (98.0) ***p*** **= 0.006**
CD4^+^/CD8^+^	2. (0.7)	2.4 (0.8) *p* = 0.240	3.8 (1.5) ***p*** **= 0.001**	2.7 (0.8) ***p*** **= 0.026**
Th-1	69.8 (25.4)	102.6 (62.4) *p* = 0.084	112.9 (61.5) ***p*** **= 0.034**	53.9 (58.9) *p* = 0.176
Th-2	64.8 (36.2)	78.5 (58.1) *p* = 0.685	89.1 (36.2) *p* = 0.060	38.5 (25.7) *p* = 0.056
Th-17	97.5 (44.0)	76.6 (27.3) *p* = 0.273	102.7 (37.6) *p* = 0.772	101.5 (52.5) *p* = 0.999
T-reg	14.8 (9.2)	11.9 (11.5) *p* = 0.465	7.4 (4.8) ***p*** **= 0.033**	14.7 (7.4) *p* = 0.983
**NK cells**	**% change** **3 months**	**% change** **6 months**	**% change** **12 months**	**% change** **24 months**
Baseline	41.2 (84.8) *n = 23,* *p = 0.346*	18.9 (80.5) *n = 29,* *p = 0.991*	75.3 (108.0) *n = 31,* ***p = 0.035***	85.9 (98.2) *n = 26,* ***p < 0.001***
3 months		−1.9 (75.0) *n = 22,* ***p = 0.045***	58.8 (98.2) *n = 18,* *p = 0.112*	50.4 (106.0) *n = 14,* *p = 0.376*
6 months			122 (213.0) *n = 21,* ***p = 0.011***	101 (200.0) *n = 17,* *p = 0.245*
12 months				31.5 (110.0) *n = 26,* *p = 0.300*

*NK cells absolute values at baseline and percentage change (SD) between different time-points during DMF therapy are also shown. p < 0.05 were considered statistically significant (bold font)*.

Within CD4^+^ T cells, Th-1 cells decrement was observed only after 2 years of treatment (Table 1). Th-17 absolute cell count showed a slight, but not significant, decrement after 6 months of therapy (Table 1). We then looked at other T cell subsets, observing a different trend in Th-2 and T-reg cells. Th-2 cell count had a slight increase after 1 year of treatment, while, after the second year of treatment, Th-2 levels resulted lower both when compared to baseline (*p* = 0.056) and one-year follow up (*p* < 0.001, Supplementary Figure 1). On the contrary, T-reg cells decreased after the first year of exposure (*p* = 0.033) and then increased, reaching values similar to baseline at the 2-years analysis (*p* = 0.983, Table 1). Percentage reflected absolute mean values changes (data not shown). Absolute B cell count showed a similar trend to CD3^+^ T cells; mean absolute count showed a decrement of 34.1% after 3 months of DMF treatment, achieving a decrement of 39.1% after 12 months (*p* < 0.001, Supplementary Table 1).

### NK Cells

Unlike T and B cells, NK absolute count showed a strong variability over the 2-years longitudinal observation. Indeed, mean absolute counts increased by 85.9% after 2 years of treatment (*p* < 0.001, Table 1), and the proportions of NK cells showed an opposite trend when compared to other lymphocyte subsets (Figure 1). However, the overall rate of such an increase was not linear, due to marked individual variability (Supplementary Figure 2). Regression analysis confirmed a positive NK longitudinal trend (average β regression coefficient of 43.1 with CV of 103) with respect to T CD3^+^ cells that showed a negative trend (average T3 β regression coefficient of −194 with CV of 73.2). This difference was statistically significant (*F*-test *p* < 0.001).

### Lymphocyte Subsets and Disease Activity

Among the 25 patients who completed the follow up, 8 showed evidence of disease activity (EDA); all of them had a new or increasing white matter T2 lesion while 5 of them also suffered a relapse.

After the two year follow up no significant differences were detected in total lymphocyte count (*p* = 0.662) in B (*p* = 1), T (*p* = 0.549) and NK cell count (*p* = 0.382) between the EDA and NEDA group.

## Discussion

In the present study, RRMS patients assigned to receive DMF treatment showed a significant decrease in total lymphocyte count since the first months of treatment. However, in line with previous studies (18, 19), a severe lymphopenia was observed only in a very small number of patients. Nevertheless, although opportunistic infections could happen also in patients without substantial lymphopenia (20), the reduction of absolute number of lymphocytes should be strictly monitored. Consistent with the trend of the total lymphocyte population, T and B cells showed an early and expected decrease in their absolute values (5, 9).

In our longitudinal study a trend of preferential decrease in CD8^+^ T cells was observed in the first year of therapy, but was not confirmed in subsequent follow up; DMF mechanism of action on T cells appears more complex than a change in main lymphocyte subsets ratio and it probably implies a shift of immune response throughout a more “tolerogenic” profile of immune system (12). Such observation was partially confirmed by the increase of T-reg cells during the second year of treatment.

Unlike T and B cell, NK cells showed a significant positive trend. The change over time appeared quite heterogeneous, with a consistent increase in percentage and a wide variance in trends of absolute cell count. With the limit of simple size, increasing percentage, and absolute count of NK cells over time did not correlate with the degree of lymphopenia (Supplementary Figure 2). Such increase was not detected in previous studies (1, 21) and could reflect an increase in immune-regulatory NK cells, particularly CD56^+bright^ cells, which was described in the first months of therapy (12). This change was correlated with reduction of CD8^+^ memory T cells and pro-inflammatory cytokine producing T cells (10) and has been associated to a low activity of the disease (12), probably related to a regulatory action on both innate and adaptive immune system of MS patients (22, 23). Although the relationship between CD56^+bright^ and other NK cell subsets is not fully understood yet (24), the mechanism by which they could regulate central nervous system inflammation in MS patients is well established (11).

Intriguingly, activation of NRF2 signaling pathway after DMF therapy has been observed to increase, among the others, the number of CD56^bright^ natural killer cells and to be related with the absence of disease activity after 1 year of therapy (25). In our patient cohort no significant differences were found in total lymphocyte count, T, B, and also NK cells in the group of patients with disease activity compared to the NEDA group. Despite the relatively long follow-up can partially balance such weakness, we are aware that our conclusions are jeopardized by the small sample size. Furthermore, the simplified characterization of T subsets, based upon surface expression of chemokine receptors that are not exclusively categorizable into a distinct subset (26), might also explain some unexpected results in their longitudinal evaluation.

Concluding, the effect of DMF on lymphocytes subsets still needs further investigations, especially with regard to NK cells count which appeared not in line with that of other lymphocytes.

## Ethics Statement

The Ethic Committee for clinical research of Azienda Sanitaria di Padova approved the present study and the informed consent was obtained from all patients.

## Author Contributions

DM, AB, RM, and MC: conception and design, acquisition, analysis and interpretation of data, drafting the paper. FC, CZ, and SM: analysis and interpretation of data, drafting the paper. AP and CR: statistical analysis and interpretation of data, drafting the paper. SF, VS, BM: conception and design and acquisition of data, drafting the paper.

### Conflict of Interest Statement

AB, SF, VS, and BM were employed by Data Medica Group, Synlab Limited, Padova, Italy. MC received payment for development of educational presentations including service on speakers bureaus by Biogen-Elan, Genzyme, TEVA, Bayer-Schering, he received travel/accomodation expenses by Novartis Pharma, Genzyme, Biogen idec, Merck Serono, Bayer-Schering, TEVA, and Advisory Board membership by Bayer-Shering, Genzyme, Biogen Idec and Novartis Pharma. The remaining authors declare that the research was conducted in the absence of any commercial or financial relationships that could be construed as a potential conflict of interest.

## Abbreviations

DMF: Dimethyl fumarate
RRMS: Relapsing Remitting Multiple Sclerosis
NK: natural killer
Nrf2: nuclear factor erythroid 2-related factor 2
MMF: Monomethyl fumarate
GAPDH: Glyceraldehyde 3-phosphate dehydrogenase
Th-1: T-helper 1
Th-17: T-helper 17
Th-2: T-helper 2
T-reg: T-regulatory
EDSS: Expanded Disability Status Scale
NEDA: No Evidence of Disease Activity
EDA: Evidence of Disease Activity.
