# Supplementary material for: Increased NK Cell Count in Multiple Sclerosis Patients Treated With Dimethyl Fumarate: A 2-Year Longitudinal Study
Source: Front Immunol. 2019 Jul 19;10:1666. doi: 10.3389/fimmu.2019.01666 (PMC6658905; doi:10.3389/fimmu.2019.01666)
Supplement: Supplementary file 1 [file Table_1.DOCX]

**Supplementary Table 1.** Absolute lymphocyte count and subsets at baseline: mean counts [x10^9^/L] and SD are reported. Percentage change of absolute count (SD) at 3, 6, 12 and 24 months when compared to baseline values is showed. *p* Values less than 0.05 were considered statistically significant.

|  | **0 months** (n=38) | **3 months** (n=23) | **6 months** (n=29) | **12 months** (n=31) | **24 months** (n=26) |
| --- | --- | --- | --- | --- | --- |
| **Lymph** | 1887.4 (533.8) | −𝟏𝟓.0 (𝟑𝟏.𝟑) *p* 0.025 | −𝟑𝟏.0 (𝟑𝟏.𝟓) *p*<0.001 | −𝟑𝟓.0 (𝟑𝟏.𝟓) *p*<0.001 | −𝟑𝟑.𝟕 (𝟑𝟓.𝟐) *p*<0.001 |
| **T cells** | 1403.7 (378.5) | −𝟏𝟔.𝟕 (𝟑𝟓.𝟔) *p* 0.016 | −𝟑5.0 (𝟑𝟒.𝟔) *p*<0.001 | −𝟒𝟕.𝟓 (𝟐𝟗.𝟐) *p*<0.001 | −𝟒𝟕.𝟕 (𝟐𝟗.𝟓) *p*<0.001 |
| **B cells** | 251.2  (171.2) | −𝟑𝟒.𝟏 (𝟐𝟗.𝟐) *p*<0.001 | −𝟑𝟑.0 (𝟒𝟑.𝟏) *p*<0.001 | −𝟑𝟗.𝟏 (𝟑𝟏.𝟒) *p*<0.001 | −𝟐𝟒.𝟖 (𝟓𝟑.𝟓) *p* 0.015 |
| **Nk cells** | 217.2  (157.2) | +𝟒𝟏.𝟐 (𝟖𝟒.𝟖) *p* 0.346 | +𝟏𝟖.𝟗 (𝟖𝟎.𝟓) *p* 0.991 | +𝟕𝟓.𝟑 (𝟏𝟎𝟖.0) *p* 0.035 | +𝟖𝟓.𝟗 (𝟗𝟖.𝟐) *p*<0.001 |
