# Supplementary figures and images for: Increased NK Cell Count in Multiple Sclerosis Patients Treated With Dimethyl Fumarate: A 2-Year Longitudinal Study
Source: Front Immunol. 2019 Jul 19;10:1666. doi: 10.3389/fimmu.2019.01666 (PMC6658905; doi:10.3389/fimmu.2019.01666)

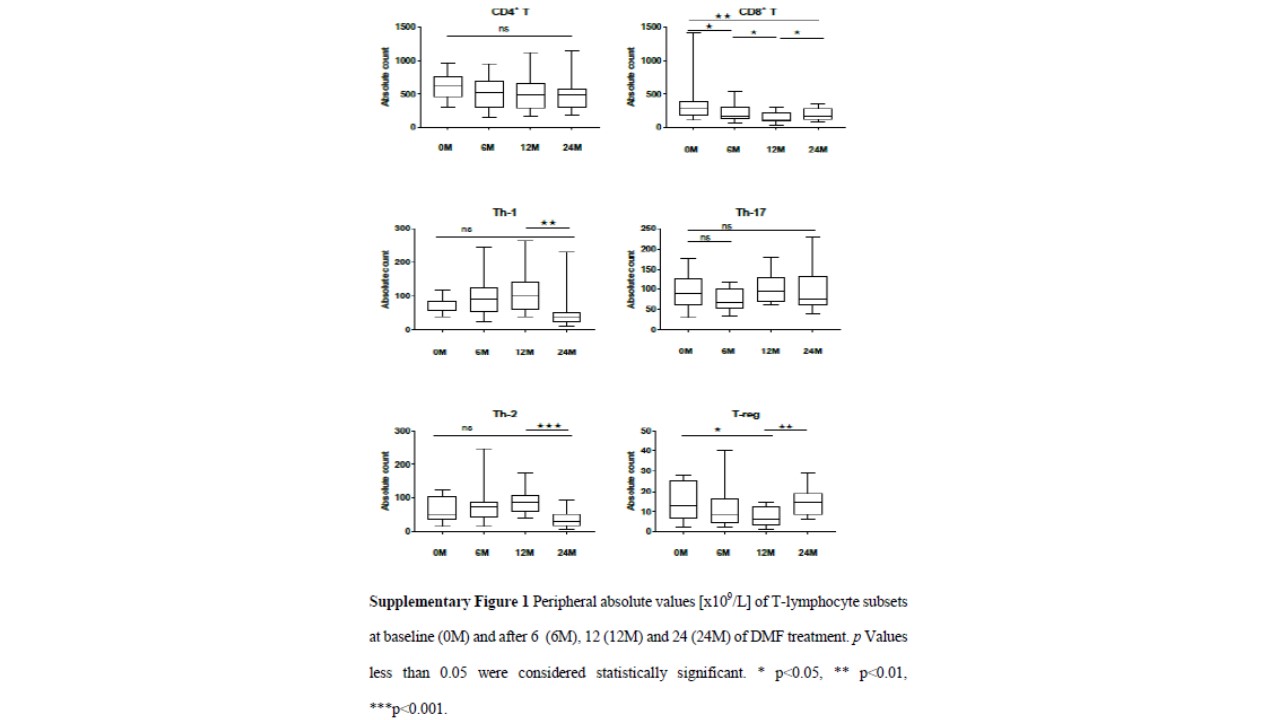

Supplement: Supplementary file 2 [file Image_1.JPEG]

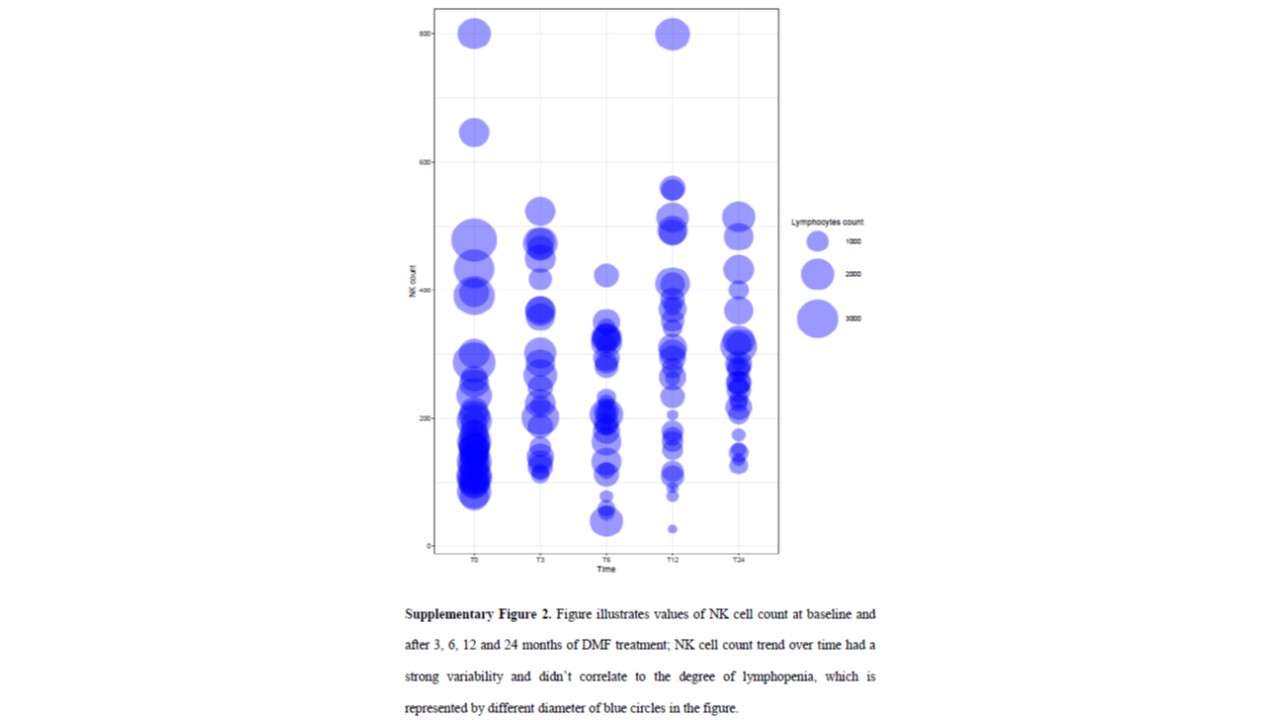

Supplement: Supplementary file 3 [file Image_2.JPEG]

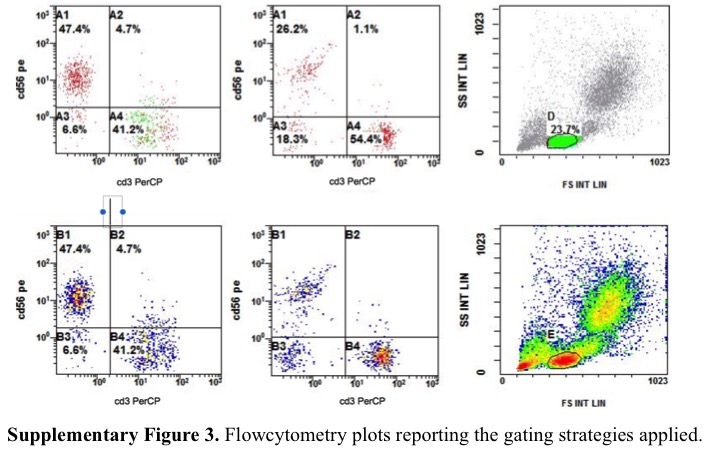

Supplement: Supplementary file 4 [file Image_3.jpg]
